# Molecular Cloning and Characterization of Four Genes Encoding Ethylene Receptors Associated with Pineapple (*Ananas comosus* L.) Flowering

**DOI:** 10.3389/fpls.2016.00710

**Published:** 2016-05-24

**Authors:** Yun-He Li, Qing-Song Wu, Xia Huang, Sheng-Hui Liu, Hong-Na Zhang, Zhi Zhang, Guang-Ming Sun

**Affiliations:** ^1^South Subtropical Crop Research Institute, Chinese Academy of Tropical Agricultural SciencesZhanjiang, China; ^2^Key Laboratory of Tropical Fruit Biology, Ministry of AgricultureZhanjiang, China; ^3^The Key Laboratory of Gene Engineering of the Ministry of Education, School of Life Sciences, Sun Yat-sen UniversityGuangzhou, China

**Keywords:** *Ananas comosus* L., ethylene, ethylene receptors, flowering, inflorescence development

## Abstract

Exogenous ethylene, or ethephon, has been widely used to induce pineapple flowering, but the molecular mechanism behind ethephon induction is still unclear. In this study, we cloned four genes encoding ethylene receptors (designated *AcERS1a*, *AcERS1b*, *AcETR2a*, and *AcETR2b*). The 5′ flanking sequences of these four genes were also cloned by self-formed adaptor PCR and SiteFinding-PCR, and a group of putative *cis*-acting elements was identified. Phylogenetic tree analysis indicated that AcERS1a, AcERS1b, AcETR2a, and AcETR2b belonged to the plant ERS1s and ETR2/EIN4-like groups. Quantitative real-time PCR showed that *AcETR2a* and *AcETR2b* (subfamily 2) were more sensitive to ethylene treatment compared with *AcERS1a* and *AcERS1b* (subfamily 1). The relative expression of *AcERS1b*, *AcETR2a*, and *AcETR2b* was significantly increased during the earlier period of pineapple inflorescence formation, especially at 1–9 days after ethylene treatment (DAET), whereas *AcERS1a* expression changed less than these three genes. *In situ* hybridization results showed that bract primordia (BP) and flower primordia (FP) appeared at 9 and 21 DAET, respectively, and flowers were formed at 37 DAET. *AcERS1a*, *AcERS1b*, *AcETR2a*, and *AcETR2b* were mainly expressed in the shoot apex at 1–4 DAET; thereafter, with the appearance of BP and FP, higher expression of these genes was found in these new structures. Finally, at 37 DAET, the expression of these genes was mainly focused in the flower but was also low in other structures. These findings indicate that these four ethylene receptor genes, especially *AcERS1b*, *AcETR2a*, and *AcETR2b*, play important roles during pineapple flowering induced by exogenous ethephon.

## Introduction

Pineapple (*Ananas comosus* L.) is one of the most popular tropical fruits. In China and many other pineapple-planting regions, one of the most serious problems of pineapple cultivation is natural flowering, which results in unscheduled fruiting (Bartholomew et al., 2003). In China, natural flowering is often observed in February after the pineapples have experienced low temperatures in January or the previous December. This natural flowering results in most of the fruits being harvested from May to June; furthermore, the natural flowering lasts for several weeks because it is not synchronized (Zhu et al., 2012), resulting in increased cultivation costs and decreased profit for pineapple growers.

Once reproductive development is initiated, pineapple inflorescence and fruit development continue without interruption until fruit maturation (Bartholomew et al., 2003). Therefore, to produce fruit in every month of the year, ethylene or ethephon (which degrades to produce ethylene) has been widely used to induce pineapple flowering (Bartholomew et al., 2003). Although there has been much research concerning the role of ethylene in flower development (Lin et al., 2009), the molecular mechanism behind ethylene induction of flower development is still unclear.

The gaseous plant hormone ethylene is an important regulator of plant growth and development, including seed germination, flower development, fruit ripening, leaf senescence, and response to environmental stress (Abeles et al., 1992; Ma et al., 2014; Wen, 2015). The transition from vegetative to reproductive growth is a key step in a plant’s life cycle. Flowering time is controlled by various endogenous and exogenous signals, including photoperiod, temperature, and plant age (Srikanth and Schmid, 2011; Song et al., 2013; van Doorn and Kamdee, 2014). Ethylene plays a role in the regulation of flowering timing, but its effects appear complicated (Ma et al., 2014). For example, in *Arabidopsis*, Ogawara et al. (2003) reported that ethylene promoted floral transition; however, the *ctr1* mutant and the 1-aminocyclopropane-1-carboxylic acid (ACC)-treated wild-type showed delayed flowering, indicating that ethylene inhibited *Arabidopsis* flowering (Achard et al., 2007). In rice, converse effects of ethylene have also been observed. Overexpression of *OsETR2* reduces ethylene sensitivity and delays floral transition, whereas the suppression of *OsETR2* by RNAi enhances ethylene sensitivity and accelerates rice flowering, indicating that ethylene promotes rice flowering (Wuriyanghan et al., 2009). By contrast, transgenic lines overexpressing *OsCTR2* and the *osctr2* loss-of-function mutant show delayed flowering, suggesting that ethylene represses the floral transition in rice (Wang et al., 2013b). On the other hand, in pineapple (*Ananas comosus*), it has been proposed that flowering is triggered by a small burst of ethylene production in the meristem in response to environmental cues. *AcACS2* was induced in the meristem during induction of the flowering, and silencing *AcACS2* in transgenic pineapple plants results in a significant flowering delay. Therefore, *AcACS2* may be the main contributor to the ethylene promotion of pineapple flowering (Trusov and Botella, 2006). These contradictory observations may be due to different growth conditions, genetic backgrounds, or mechanisms employed by these signaling components (Ma et al., 2014).

A linear signaling pathway has been established on the basis of genetic analyses of ethylene-responsive *Arabidopsis* mutants (Ma et al., 2014). In this pathway, the first step is the binding of ethylene to the ethylene receptors, which play a crucial role by negatively regulating ethylene responses (Chang and Stadler, 2001). In *Arabidopsis thaliana*, five ethylene receptor genes have been identified, including *ETR1*, *ETR2*, *ERS1*, *ERS2*, and *EIN4* (Hua et al., 1998; Schaller and Kieber, 2002; Chen et al., 2005; O’Malley et al., 2005; Kendrick and Chang, 2008). Based on phylogenetic analysis, the ethylene receptors can be divided into two subfamilies that share structural features: subfamily 1 has three hydrophobic regions plus a conserved histidine kinase domain and includes ETR1 and ERS1; whereas subfamily 2 has four hydrophobic regions and a more diverged kinase domain and includes ETR2, ERS2, and EIN4 (Chang and Stadler, 2001; Schaller and Kieber, 2002; Chen et al., 2005).

In the absence of the ethylene, receptors actively suppress ethylene responses; when ethylene binds, the suppression is removed and the response occurs (Wen, 2015). All five *Arabidopsis* receptors are involved in ethylene signaling and have overlapping roles in regulating ethylene responses (Hua et al., 1995, 1998; Hall and Bleecker, 2003). However, recent studies showed that the five receptor isoforms are not entirely redundant in their roles (Hall and Bleecker, 2003; Binder et al., 2004, 2006; Seifert et al., 2004; O’Malley et al., 2005; Xie et al., 2006; Kevany et al., 2007; Qu et al., 2007; Plett et al., 2009a,b; Liu et al., 2010; Wang et al., 2013a; Wilson et al., 2014). For instance, only specific ethylene receptors mediate fruit ripening in tomato, but other receptors had little or no effect on fruit ripening (Tieman et al., 2000; Kevany et al., 2007). In addition, the *etr1 ers1* mutant phenotype can be rescued by the expression of subfamily 1 receptors but not subfamily 2 receptors, which indicated the unique function for subfamily 1 receptors in *Arabidopsis* (Wang et al., 2013a).

Ethylene receptors are involved in many biological processes, such as ethylene-induced nutational bending of the apical hook, recovery of growth after ethylene treatment, nutational bending, control of flowering time and fruit maturation and response to salt stress (Merchante et al., 2013; Shakeel et al., 2013). Ethylene receptor genes can be induced by various abiotic stresses, including wounding, salt and drought treatments (Zhang et al., 1999; Zhang J. et al., 2001; Zhang J.S. et al., 2001; Xie et al., 2002; Tao et al., 2015), and ethylene receptors also regulate plant defense responses (He et al., 2005; Cao et al., 2007; Wilson et al., 2014; Tao et al., 2015). For example, under salt stress, loss of ETR1 or EIN4 promotes *Arabidopsis* seed germination, loss of ETR2 delays germination, and loss of either ERS1 or ERS2 has no measurable effect on germination (Wilson et al., 2014). In addition to stress responses, ethylene receptors are highly expressed in reproductive organs and may be involved in reproductive development (Hua et al., 1998; Tieman et al., 2000; Yamasaki et al., 2000; Zhang J. et al., 2001; Zhang J.S. et al., 2001; Klee, 2002, 2004; Xie et al., 2002; Rieu et al., 2003; Zhou et al., 2006; Wuriyanghan et al., 2009; Thongkum et al., 2015). For instance, reduction of the expression of the tomato ethylene receptors LeETR4 or LeETR6 increased ethylene production and promoted ripening (Tieman et al., 2000; Kevany et al., 2007), which means that climacteric fruit ripening is also controlled at the receptor level (Lin et al., 2009).

Here, we reported the isolation and characterization of four genes encoding ethylene receptors in pineapple and their promoters. Using two different treatment concentrations of ethephon, we studied the genes’ temporal and spatial expression during inflorescence induction by quantitative real-time PCR (qRT-PCR) and *in situ* hybridization.

## Materials and Methods

### Plant Materials and Treatments

Pineapples (*Ananas comosus* L. cv. Perola) were planted in the South Subtropical Crop Research Institute, at Zhanjiang, Guangdong province, China (20°39′ N latitude and 110°15′ E longitude). Homogenous 15-month-old plants were selected for induced flowering. In September 2012, after 5 pm in the evening, 50 ml of 200 or 1200 mg l^-1^ ethephon (5 or 30 ml of 40% ethephon diluted to 10 l with water, respectively) was poured over each group of plants; the control plant group was treated with 50 ml of water. Each treatment group and the control group contained at least 300 plantings. The two different concentrations of ethephon were selected because our previous study (Liu et al., 2009) showed that the application of 200 and 800 mg l^-1^ ethephon induced 100% pineapple (‘Zhenzhu’) flowering, but the higher ethephon concentration resulted in smaller fruits.

After ethephon treatment, the shoot apex or infiorescence of the pineapple was collected at 0, 1, 2, 5, 9, 14, 21, 28, and 37 days and either immediately frozen in liquid nitrogen followed by storage at -80°C until RNA extraction or fixed for subsequent *in situ* hybridization.

### RT-PCR with Degenerate Primers and Cloning of PCR Products

After 2 days of ethylene treatment, the shoot apex was collected and was used to extract total RNA using the method of Fu et al. (2004). cDNA was synthesized from 2 μg of RNA using PrimeScript II first Strand cDNA Synthesis Kit (Takara Bio Inc., Dalian, China) and Oligo dT as a primer, following the manufacturer’s instructions.

This cDNA was used as a template to isolate DNA fragments encoding ethylene receptors by PCR. We used five pairs of degenerate primers (**Supplementary Table S1**) designed from the alignment of sequences from other plants in the program iCODEHOP v1.1 (Boyce et al., 2009). The conditions of the PCR were as follows: 94°C for 3 min, 40 cycles at 94°C for 30 s, 54°C for 1 min, and 72°C for 2 min and a final 7 min extension at 72°C using a DNA thermal cycler. The purified products were cloned into the pMD-18 T vector (Takara Bio Inc., Dalian, China) and sequenced by the Invitrogen Company (Guangzhou, China) using an ABI 3770 DNA sequencer.

### Cloning of *AcERS1a*, *AcERS1b*, *AcETR2a*, and *AcETR2b* Full-Length cDNAs by RACE

From the initial partial cDNA fragments, five full-length ethylene receptor cDNA clones were produced by 5′ and 3′ RACE. The RACE reactions were performed using the SMARTer^TM^ RACE cDNA Amplification Kit (Clontech) according to the manufacturer’s instructions.

To obtain the 5′ cDNA regions of the ethylene receptor genes, the primer sets AcETR1SP1 and UPM, AcERS1SP1 and UPM, AcETR2SP1 and UPM, AcEIN4SP1 and UPM, and AcERS2SP1 and UPM were used for the first-round PCR of the 5′ RACE for *AcETR1*, *AcERS1*, *AcETR2*, *AcEIN4*, and *AcERS2*, respectively. The primer sets AcETR1SP2 and NUP, AcERS1SP2 and NUP, AcETR2SP2 and NUP, AcEIN4SP2 and NUP, and AcERS2SP2 and NUP were then used for nested PCR. To obtain the 3′ cDNA regions of the ethylene receptor genes, the primer sets AcETR1SP3 and UPM, AcERS1SP3 and UPM, AcETR2SP3 and UPM, AcEIN4SP3 and UPM, and AcERS2SP3 and UPM were used for the first-round PCR of the 3′ RACE. The primer sets AcETR1SP2 and NUP, AcERS1SP2 and NUP, AcETR2SP2 and NUP, AcEIN4SP2 and NUP, and AcERS2SP2 and NUP were then used for nested PCR. The conditions for the first-round 5′ RACE and 3′ RACE PCR experiments were as described by Li et al. (2012), and primers listed in **Supplementary Table S1**.

The sequencing and BLAST results showed that the cDNAs from the AcERS2 primer set and the AcEIN4 primer set were the same; they are typical undifferentiated ETR2/EIN4-type receptors found throughout monocots and more similar to ETR2 than EIN4 (Gallie, 2015), therefore both were designated *AcETR2b*. In addition, the cDNA clone from the AcETR1 primer set belonged to *ERS1*, not *ETR1*, so it was designated *AcERS1b*; the cDNA clone from AcERS1 was designated *AcERS1a*.

According to the 5′ RACE and 3′ RACE results, the specific primer sets AcERS1aSP5 and AcERS1aSP6, AcERS1bSP5 and AcERS1bSP6, AcETR2aSP5 and AcETR2aSP6, and AcETR2bSP5 and AcETR2bSP6 (**Supplementary Table S1**) were used to obtain the full-length cDNAs of *AcERS1a*, *AcERS1b*, *AcETR2a*, and *AcETR2b*, respectively. The full-length cDNAs were amplified via PCR using the 5′ RACE-Ready cDNA as the pineapple template, the conditions of the PCR were as described by Li et al. (2012). The PCR products were purified and cloned into the pMD-18T vector and sequenced.

### Isolation of the *AcERS1a*, *AcERS1b*, *AcETR2a*, and *AcETR2b* Genes from Pineapple Genomic DNA

Genomic DNA from pineapple leaves was extracted using the Plant DNAout kit (Tiandz, Beijing, China) following the manufacturer’s instructions. The primer sets are listed in **Supplementary Table S1**. The specific primers sets AcERS1aSP5 and AcERS1aSP6, AcERS1bSP5 and AcERS1bSP6, AcETR2aSP5 and AcETR2aSP6, and AcETR2bSP5 and AcETR2bSP6 (**Supplementary Table S1**), were used to amplify the *AcERS1a*, *AcERS1b*, AcET*R2a*, and *AcETR2b* genes, respectively, from pineapple genomic DNA.

PCR amplification was carried out under the following conditions: 3 min at 94°C, followed by 30 cycles of 10 s at 98°C and 8 min at 68°C. The purified products were cloned into the pMD-18T vector and sequenced.

### Promoters Isolation

Self-formed adaptor PCR (SEFA PCR; Wang et al., 2007) was used to isolate the promoter of *AcERS1a*, while SiteFinding-PCR (Tan et al., 2005) was used to isolate the promoters of *AcERS1b*, *AcETR2a*, and *AcETR2b*. Genomic DNA from pineapple leaves was extracted as above. Based on the genomic DNA sequences of *AcERS1a*, *AcERS1b*, *AcETR2a*, and *AcETR2b*, primers for use in SEFA PCR and SiteFinding-PCR were designed (listed in **Supplementary Table S1**). The PCR reactions were performed as described by Tan et al. (2005) and Wang et al. (2007) with slight modifications. In the SEFA PCR, only three primers were used in the PCR (i.e., AcERS1a-GSP1, AcERS1a-GSP2, and AcERS1a-GSP3); for the SiteFinding-PCR, long Taq DNA polymerase was purchased from Takara (Dalian, China), and GSP3 was not used in the PCR. The purified products were cloned into the pMD-18T vector and sequenced.

### Bioinformatics Analysis

The nucleotide sequences, deduced amino acid sequences, and open reading frames (ORFs) were analyzed online^1^. The genomic DNA of the five *Arabidopsis thaliana* ethylene receptor genes was obtained from TAIR^2^. The deduced amino acid sequences were compared using BLAST against the NCBI’s Conserved Domain Database (CDD)^3^ to find the conserved domains. MW and pI predictions for the deduced AcERS1a, AcERS1b, AcETR2a, and AcETR2b oligopeptides were carried out with the Compute pI/MW tool^4^. Sequence alignment was performed using ClustalW^5^. The ClustalW-produced alignment file was formatted using the BOXSHADE program^6^. Phylogenetic analysis and construction of a neighbor-joining tree were performed using the MEGA 4.0 software using the bootstrap method with 1,000 bootstrap iterations. Putative cis-acting elements upstream of the start codons of *AcERS1a*, *AcERS1b*, *AcETR2a*, and *AcETR2b* were identified by searching the PLACE databases^7^ (Higo et al., 1999).

### Quantitative Real-Time PCR

*AcERS1a*, *AcERS1b*, *AcETR2a*, and *AcETR2b* transcript levels were determined by quantitative real-time PCR. Total RNA was extracted using the method of Fu et al. (2004). First-strand cDNA was prepared from 1 μg of total RNA with oligo dT primers using the PrimeScript RT reagent Kit with gDNA Eraser (Takara Bio Inc., Dalian, China) according to the manufacturer’s protocol and stored at -20°C. Real-time PCR was performed using the Thermo Scientific DyNAmo ColorFlash SYBR Green qPCR Kit (Thermo) on a Stratagene Mx3005P quantitative PCR machine. The following program was used: 95°C for 7 min, followed by 40 cycles of 95°C for 5 s, 56°C for 15 s, and 72°C for 25 s. As an internal control, levels of *actin* were quantified in parallel with the target genes (Li et al., 2012). Normalization and fold changes were calculated using the ΔΔ*C*t method, three independent biological replicates were used in the analysis. Primer sets are listed in **Supplementary Table S2**.

### *In Situ* Hybridization

Samples were fixed in formaldehyde and acetic acid (50% ethanol, 5% acetic acid, and 3.7% formaldehyde) for 24 h at 4°C, and the hybridization and immunological detection steps were performed as described by Dai et al. (2007). Nine-micrometer thick sample sections were cut with a microtome; all of the sections in this study were cut longitudinally. The *AcERS1a*, *AcERS1b*, *AcETR2a*, and *AcETR2b* probes were amplified using gene-specific primers (**Supplementary Table S2**). The PCR fragments were inserted into the NcoI and SalI sites of pGE-T (Tiangen, China) and transcribed *in vitro* from either the T7 or SP6 promoter for sense or antisense strand synthesis, respectively, using the Digoxigenin RNA Labeling kit (Roche).

### Subcellular Localization

To analyze the subcellular localization, the ORFs of *AcERS1a*, *AcERS1b*, *AcETR2a*, and *AcETR2b* without termination codon were obtained by PCR amplification using specific primers (**Supplementary Table S3**), and subsequently cloned into the pGFP2 vector at the XhoI/KpnI sites using In-Fusion HD Cloning Kits (Clontech) according to the manufacturer’s instructions, which resulted in the 35S::gene-GFP vectors under the control of the CaMV 35S promoter. The fusion constructs and the control GFP vector were transformed into tobacco BY2 protoplasts by PEG method as described by Shan et al. (2012). GFP fluorescence was observed with a fluorescence microscope (Zeiss Axioskop 2 Plus).

### Statistical Analysis

The data were analyzed using analysis of variance (ANOVA) followed by Duncan’s multiple range test or an independent-samples *t*-test at a 5% significance level.

## Results

### Effect of Different Concentrations of Ethephon on Pineapple Flowering and Fruiting

Flowering was observed approximately 2.5 months after ethephon treatment; however, at that time, none of the control plants were flowering. The control plants flowered naturally during the following February. A previous study showed that higher ethephon concentrations inhibited the growth of ‘Zhenzhu’ pineapple fruits and that smaller fruits were harvested (Liu et al., 2009). However, for ‘Perola’ pineapples, 200 or 1200 mg l^-1^ ethephon had no effect on the flowering rate (both were 100%), flowering time (the time to the first flower appearing after ethylene treatment), or fruit qualities, such as fruit weight and number of fruitlets (**Table 1**). The different reactions to higher ethephon concentrations may be due to the different sensitivities to ethephon treatment between ‘Perola’ and ‘Zhenzhu’ pineapples. Therefore, we studied only the ethylene receptor genes’ *in vivo* transcript profiles for the 200 mg l^-1^ ethephon treatment.

**Table 1:** Effect of different concentrations of ethephon on pineapple flowering time and fruit weight.

Ethephon concentration (mg l^-1^)	Flowering rate	Flowering time (DAET)	Fruitlet number	Fruit weight (g)	Fruit weight without crown (g)
Control	0%	–	–	–	–
200	100%	93.5 ± 0.3	81.4 ± 1.5	643.3 ± 11.7	509.6 ± 11.4
1200	100%	94.4 ± 0.4	78.2 ± 1.0	661.1 ± 12.9	507.5 ± 13.4
Significance	NS	NS	NS	NS	NS

*Values represent the means ± standard error (SE) with at least 100 plants or fruits per treatment. Data in the same column were analyzed using an independent-samples t-test. DAET, days after ethephon treatment; NS, not significant.*

### Isolation and Characterization of the cDNA Clones of *AcERS1a*, *AcERS1b*, *AcETR2a*, and *AcETR2b* from Pineapple

Using different primer sets (**Supplementary Table S1**), five different cDNA fragments were obtained from RT-PCR, and using RACE extension, five full-length cDNA clones of pineapple ethylene receptor genes were obtained. However, the sequencing and BLAST results showed that the cDNAs from the AcERS2 primer set and the AcEIN4 primer set were the same and did not contain the conserved histidine in the H-motif; therefore, they belong to the undifferentiated ETR2/EIN4 group and are more similar to ETR2 than EIN4 (Gallie, 2015). Both were designated *AcETR2b* (the cDNA clone from the AcETR2 primer set was designated *AcETR2a*). On the other hand, the BLAST results showed that the cDNA clone from the AcETR1 primer set belonged to *ERS1* and not *ETR1*, so it was designated *AcERS1b*. The cDNA clone from AcERS1 was designated *AcERS1a*.

The corresponding full-length *AcERS1a*, *AcERS1b*, *AcETR2a*, and *AcETR2b* cDNA clones were 2304, 2411, 2558, and 2635 bp long and contained ORFs of 1938, 1905, 2268, and 2277 bp, respectively. The *AcERS1a*, *AcERS1b*, *AcETR2a*, and *AcETR2b* genes encode a putative protein of 645, 634, 755, and 758 amino acids with a predicted MW of 72.11, 71.28, 83.19, and 84.53 kDa and a pI of 6.39, 6.77, 8.71, and 6.67, respectively. The sequences of *AcERS1a*, *AcERS1b*, *AcETR2a*, and *AcETR2b* have been submitted to the GenBank database (accession numbers KM062066, KM062063, KM062064, and KM062065, respectively).

**FIGURE 1:**
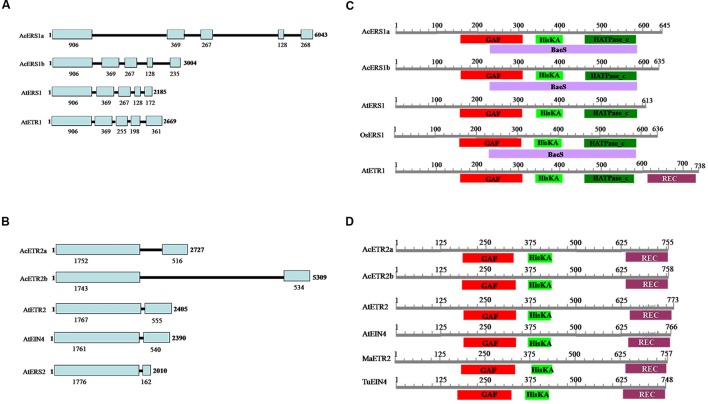
**Pineapple (*Ananas comosus* L.) ethylene receptors. (A)** Comparison of the genomic structures of *AcERS1a* (KR610528) and *AcERS1b* (KR610529) with *AtETR1* (TAIR: AT1G66340) and *AtERS1* (TAIR: AT2G40940). **(B)** Comparison of the genomic structures of *AcETR2a* (KR610530) and *AcETR2b* (KR610531) with *AtETR2* (TAIR: AT3G23150), *AtEIN4* (TAIR: AT3G04580) and *AtERS2* (TAIR: AT1G04310). **(C)** Domain structures of AcERS1a, AcERS1b, and other ERS1s and ETR1 proteins. **(D)** Domain structures of AcETR2a, AcETR2b, and other ETR2 and EIN4 proteins. The sequences used in the domain structures were obtained from the GenBank databases: AcERS1a (AIT52526), AcERS1b (AIT52523), AcETR2a (AIT52524), and AcETR2b (AIT52525) from *Ananas comosus* (Ac); AtERS1 (AAC49090), AtETR1 (AAA70047), AtETR2 (AAC62208), and AtEIN4 (AAD02485) from *Arabidopsis thaliana* (At); MaETR2 (XP_009386224) from *Musa acuminata* AAA Group (Ma); OsERS1 (AAB72193), from *Oryza sativa* (Os); and TuEIN4 (EMS56298) from *Triticum urartu* (Tu).

The lengths of the genomic DNA sequences of *AcERS1a*, *AcERS1b*, *AcETR2a*, and *AcETR2b* are shown in **Figure 1**. Comparisons between the cDNA sequences and the genomic sequences revealed that the genomic DNA lengths of *AcERS1a*, *AcERS1b*, and *AtERS1* were different, but their genomic DNA structures were similar, all had five exons and four introns in their genomic DNA (**Figure 1A**). Similarly, although the genomic DNA lengths of *AcETR2a* and *AcETR2b* were different, both had two exons and one intron (**Figure 1B**). The corresponding 5′ untranslated regions (UTRs) of *AcERS1a*, *AcERS1b*, *AcETR2a*, and *AcETR2b* were 1900, 1101, 32, and 122 bp, respectively. When the UTRs were compared with the genes’ cDNAs, there were 1734 and 882 bp-long introns located in the genes’ 5′ UTRs located 40 and 41 bp from the start codons of the *AcERS1a* and *AcERS1b* genes, respectively.

Homology searches were performed with the BLAST and BLASTX algorithms to confirm sequence identity. The results indicated that the deduced amino acid sequence of *AcERS1a* and *AcERS1b* shared a high sequence identity with the ETR1 and ERS1 proteins of diverse plant species, and AcETR2a and AcETR2b shared high sequence identity with the genes encoding ETR2s or EIN4s of diverse plant species.

A search of the CDD (Marchler-Bauer et al., 2015) for conserved protein domains indicated that both the predicted amino acid sequences of *AcERS1a* and *AcERS1b* contained a GAF domain, a HisKA (histidine kinase A) domain, a HATPase_c (histidine kinase-like ATPase) domain, and a BaeS (signal transduction histidine kinase) domain. These four domains are also found in OsERS1 (AAB72193), but the BaeS domain is not found in AtERS1 and AtETR1 (**Figure 1C**, **Supplementary Figure S1**).

Similarly, a search of the CDD for conserved protein domains indicated that both AcETR2a and AcETR2b contained a GAF domain, a HisKA domain and a REC (signal receiver domain) domain, which was similar to MaETR2, AtETR2, and AtEIN4 (**Figure 1D**; **Supplementary Figure S1**).

**FIGURE 2:**
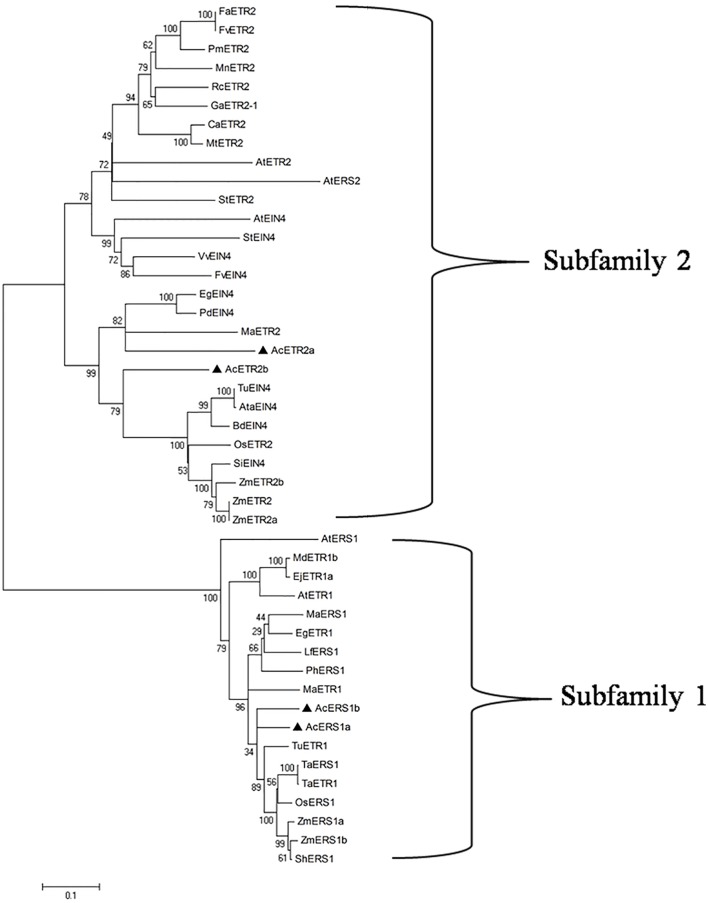
**Phylogenetic analysis of ethylene receptors.** The phylogenetic tree of the ethylene receptor proteins. Using the ClustalW program, the tree was constructed using the neighbor-joining method after sequence alignment. The scale bar corresponds to 0.1 amino acid substitutions per residue. The sequences used in this alignment were obtained from the GenBank databases: AcERS1a (AIT52526), AcERS1b (AIT52523), AcETR2a (AIT52524), and AcETR2b (AIT52525) from *Ananas comosus* (Ac); AtERS1 (AAC49090), AtETR1 (AAA70047), AtETR2 (AAC62208), AtEIN4 (AAD02485), and AtERS2 (AAC62209) from *Arabidopsis thaliana* (At); AtaEIN4 (EMT13419) from *Aegilops tauschii* (Ata); BdEIN4 (XP_003580931) from *Brachypodium distachyon* (Bd); CaETR2 (XP_004495309) from *Cicer arietinum* (Ca); EgETR1 (XP_010914501) and EgEIN4 (XP_010935766) from *Elaeis guineensis* (Eg); EjETR1a (AGE15296) from *Eriobotrya japonica* (Ej); FaETR2 (CAC48386) from *Fragaria × ananassa* (Fa); FvETR2 (XP_004288459) and FvEIN4 (XP_004302073) from *Fragaria vesca* subsp. Vesca (Fv); GaETR2-1 (AGG55710) from *Gossypium arboretum* (Ga); LfERS1 (ABD66593) from *Lilium formosanum × Lilium longiflorum* (Lf); MaERS1 (BAF44104) and MaETR2 (XP_009386224) from *Musa acuminata* AAA Group (Ma); MdETR1b (AAW69924) from *Malus domestica* (Md); MnETR2 (EXB98165) from *Morus notabilis* (Mn); MtETR2 (KEH42695) from *Medicago truncatula* (Mt); OsERS1 (AAB72193) and OsETR2 (AAN15203) from *Oryza sativa* (Os); PdEIN4 (XP_008787409) from *Phoenix dactylifera* (Pd); PhERS1 (AAD04949) from *Phalaenopsis hybrid* cultivar (Ph); PmETR2 (XP_008224393) from *Prunus mume* (Pm); RcETR2 (XP_002529316) from *Ricinus communis* (Rc); ShERS1 (ADJ66722) from *Saccharum hybrid* cultivar ROC20 (Sh); SiEIN4 (XP_004975078) from *Setaria italica* (Si); StETR2 (XP_006350680) and StEIN4 (XP_006345886) from *Solanum tuberosum* (St); TaERS1 (ADJ67795) and TaETR1 (AHL44971) from *Triticum aestivum* (Ta); TuETR1 (EMS66524) and TuEIN4 (EMS56298) from *Triticum urartu* (Tu); VvEIN4 (XP_002270757) from *Vitis vinifera* (Vv); and ZmERS1a (AFW67901), ZmERS1b (DAA50871), ZmETR2 (NP_001104852), ZmETR2a (AY359580), and ZmETR2b (AY359581) from *Zea mays* (Zm).

To evaluate the molecular evolutionary relationships of ethylene receptors in other species, we retrieved 46 different ethylene receptor proteins from several plant species through BLASTP searches, and a phylogenetic tree was constructed using the neighbor-joining method. As shown in **Figure 2**, phylogenetic analysis based on multiple alignment showed that there were two distinct subfamilies: AcERS1a and AcERS1b fell into a cluster with AtERS1 and AtETR1 (subfamily 1), whereas AcETR2a and AcETR2b fell into the other cluster with AtETR2, AtEIN4, and AtERS2 (subfamily 2).

### Isolation and Identification of the Promoters

Using SEFA PCR (Wang et al., 2007), we isolated a 5114-bp fragment upstream of the start codon of *AcERS1a*, and using SiteFinding-PCR (Tan et al., 2005), we isolated 3502-bp, 1592-bp and 1149-bp fragments upstream of the start codon of *AcERS1b*, *AcETR2a*, and *AcETR2b* (promoter sequences listed in the **Supplementary Figure S2**).

Using the PLACE web tools, several putative cis-regulatory elements were deciphered from the promoter sequences of *AcERS1a*, *AcERS1b*, *AcETR2a*, and *AcETR2b*. The types of cis-regulatory elements between *AcERS1a* and *AcERS1b* were similar, and their differences were mostly in the number of repeats (**Supplementary Table S4**). TATA box sequence elements, which are required for critical and precise transcription initiation, were found in large numbers in all promoters. Several other types of regulatory elements were found in the four promoters, including cis-acting elements involved in the response to ethylene, cytokinin and auxin (**Supplementary Table S4**). In general, more *cis*-regulatory elements and repeats were found in the promoter regions of *AcERS1a* and *AcERS1b* than of *AcETR2a* and *AcETR2b*; the detailed comparisons of the putative cis elements in these promoters are listed in **Supplementary Table S4**.

### Expression of *AcERS1a*, *AcERS1b*, *AcETR2a*, and *AcETR2b* during Pineapple Flowering Formation

For the control, the relative expression levels of *AcERS1a*, *AcERS1b*, *AcETR2a*, and *AcETR2b* were similar; none significantly changed during the whole period. However, for the ethylene treatment groups, the expression pattern of *AcERS1a* was different from the other three genes.

**FIGURE 3:**
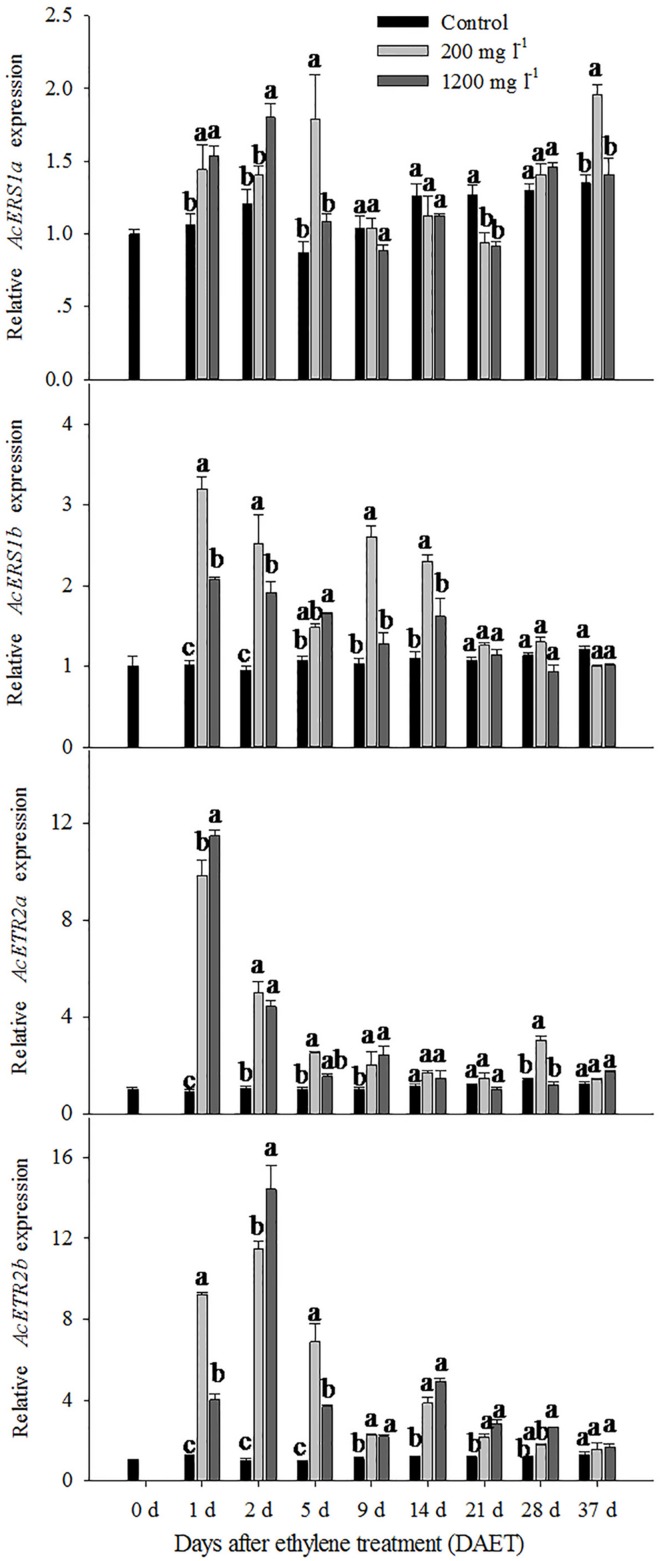
**The expression of *AcERS1a*, *AcERS1b*, *AcETR2a*, and *AcETR2b* during pineapple (*Ananas comosus*) flower transition.** The expression of *AcERS1a*, *AcERS1b*, *AcETR2a*, and *AcETR2b* was evaluated using quantitative RT-PCR (qRT-PCR). Expression levels were normalized using actin as the endogenous control gene. The values represent the means ± SE from three independent experiments. For each gene, values within every time point followed by the same letter are not significantly different (*P* > 0.05) between treatments according to Duncan’s multiple range test.

For the 200 mg l^-1^ ethylene treatment, the relative expression levels of *AcERS1a* were up-regulated compared with the control at 1 DAET (less than 1.5-fold), 5 DAET (approximately 2-fold) and 37 DAET (approximately 1.5-fold; **Figure 3**). The 1200 mg l^-1^ ethylene treatment response was similar to the 200 mg l^-1^ ethylene treatment. Compared with the control, the relative expression levels of *AcERS1a* were up-regulated at 1 and 2 DAET (less than 1.5-fold) when treated with 1200 mg l^-1^ ethylene (**Figure 3**).

The expression pattern of *AcERS1b* was different than *AcERS1a*. For the 200 and 1200 mg l^-1^ ethylene treatment, the relative expression level of *AcERS1b* was significantly (*p* < 0.05) up-regulated at 1 DAET and remained up-regulated until 14 DAET. Then, the relative expression level decreased sharply and showed similar expression to the control after 21 DAET. During this period, the relative change in expression level of 1200 mg l^-1^ was lower than the treatment with 200 mg l^-1^, except at 5 DAET (**Figure 3**).

Compared with *AcERS1a* and *AcERS1b* (subfamily 1), *AcETR2a* and *AcETR2b* (subfamily 2) were more sensitive to ethylene treatment (**Figure 3**). For the 200 mg l^-1^ and 1200 mg l^-1^ ethylene treatments, the relative expression levels of *AcETR2a* were similar; both were sharply up-regulated and reached their peaks at 1 DAET (10.9-fold and 12.7-fold, respectively). Thereafter, the expression levels decreased but remained significantly up-regulated (*p* < 0.05) at 2–9 DAET compared with the control (**Figure 3**).

Both the 200 and 1200 mg l^-1^ ethylene treatments significantly up-regulated (*p* < 0.05) the relative expression levels of *AcETR2b* from 1 to 21 DAET compared with the control. The expression of the 1200 mg l^-1^ ethylene treatment peaked at 2 DAET, but the expression of the 200 mg l^-1^ treatment peaked at 1 day (**Figure 3**).

### *In Situ* Hybridization of *AcERS1a*, *AcERS1b*, *AcETR2a*, and *AcETR2b*

To study the spatial expression of *AcERS1a*, *AcERS1b*, *AcETR2a*, and *AcETR2b* during pineapple inflorescence induction, we performed *in situ* hybridization using the Digoxigenin RNA Labeling kit. Based on the results of **Table 1**, we only studied the *in vivo* transcripts profiles of the 200 mg l^-1^ ethephon treatment group.

**FIGURE 4:**
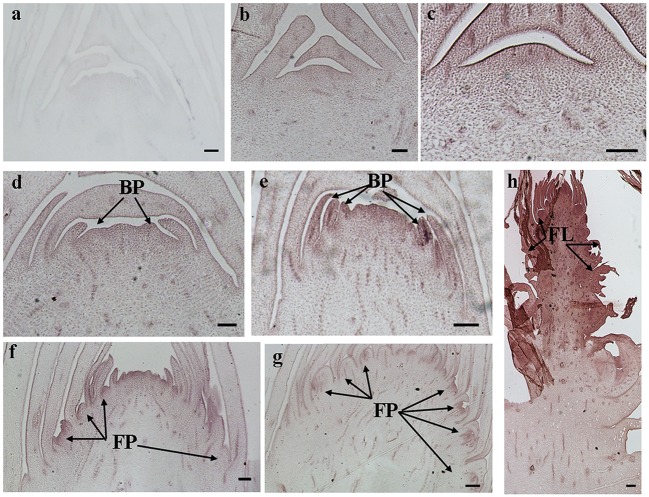
***In situ* detection of *AcERS1a* transcripts. (a–h)** Longitudinal sections of shoot apices and inflorescences sampled at 9 and 1, 5, 9, 14, 21, 28, and 37 days after ethephon treatment, respectively. Sections were hybridized with *AcERS1a* antisense **(b–h)** or sense **(a)** probes. BP, bract primordia; FP, flower primordia; FL, flower. Bars = 100 μm.

**FIGURE 5:**
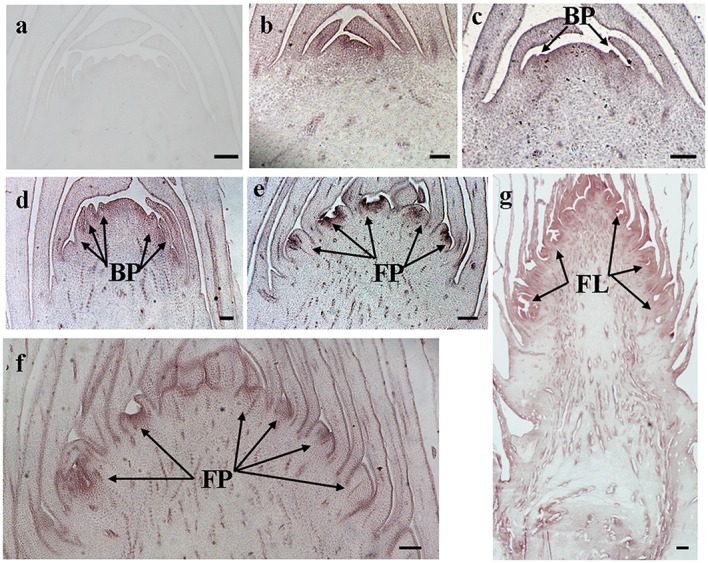
***In situ* detection of *AcERS1b* transcripts. (a–g)** Longitudinal sections of shoot apices and inflorescences sampled at 14 and 1, 9, 14, 21, 28, and 37 days after ethephon treatment, respectively. Sections were hybridized with *AcERS1b* antisense **(b–g)** or sense **(a)** probes. BP, bract primordia; FP, flower primordia; FL, flower. Bars = 100 μm.

**FIGURE 6:**
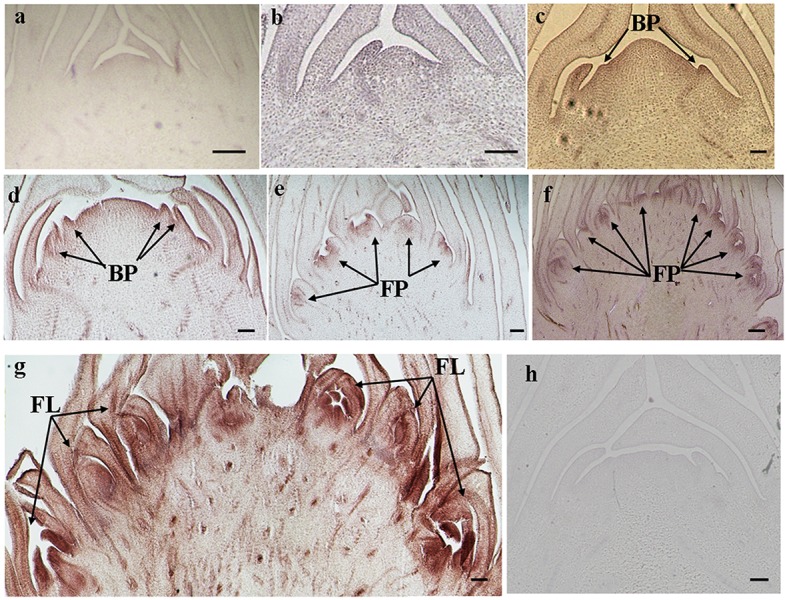
***In situ* detection of *AcETR2a* transcripts. (a–h)** Longitudinal sections of shoot apices and inflorescences sampled at 0 and 5, 9, 14, 21, 28, 37, and 9 days after ethephon treatment, respectively. Sections were hybridized with *AcETR2a* antisense **(a–g)** or sense **(h)** probes. BP, bract primordia; FP, flower primordia; FL, flower. Bars = 100 μm.

**FIGURE 7:**
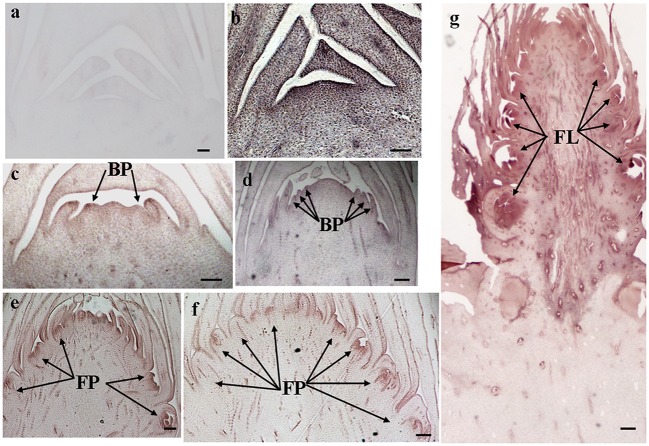
***In situ* detection of *AcETR2b* transcripts. (a–g)** Longitudinal sections of shoot apices and inflorescences sampled at 0 and 1, 9, 14, 21, 28, and 37 days after ethephon treatment, respectively. Sections were hybridized with *AcETR2b* antisense **(b–g)** or sense **(a)** probes. BP, bract primordia; FP, flower primordia; FL, flower. Bars = 100 μm.

The histological results showed there was no new structure development until 5 DAET. At least two BP were observed (**Figures 4d**, **5c**, **6c**, and **7c**) at 9 DAET, and more BP developed at 14 DAET. From 21 to 28 DAET, FP developed, each located between two bracts (**Figures 4f,g**, **5e,f**, **6e,f**, and **7e,f**). Finally, at 37 DAET, flowers were formed at the inflorescence (**Figures 4h**, **5g**, **6g**, and **7g**).

The spatial expression patterns of *AcERS1b*, *AcETR2a*, and *AcETR2b* were similar, but *AcERS1a* was lower, especially in the later period. For the earliest stages after ethephon treatment (from 0–5 DAET), *AcERS1a*, *AcERS1b*, *AcETR2a*, and *AcETR2b* were mainly expressed at the shoot apex. Following the appearance of BP and FP, higher expression of these genes was found in these new structures. When the flower formed at 37 DAET, these genes were mostly expressed in the flower, with minor expression in other locations (**Figures 4–7**).

### Subcellular Localization of AcERS1a, AcERS1b, AcETR2a, and AcETR2b

**FIGURE 8:**
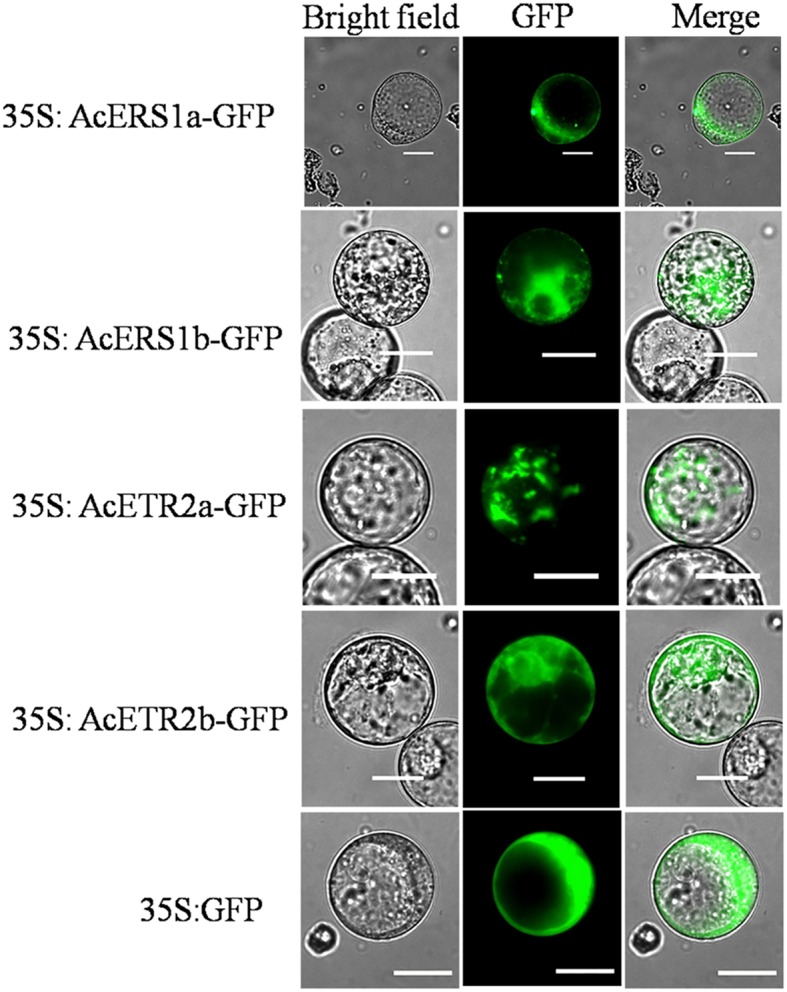
**Subcellular localization of AcERS1a, AcERS1b, AcETR2a, and AcETR2b in tobacco BY-2 protoplasts.** Protoplasts were transiently transformed with AcERS1a, AcERS1b, AcETR2a, and AcETR2b–GFP constructs or GFP vector using a modified PEG method. GFP fluorescence was observed with a fluorescence microscope. Bars = 25 μm.

To examine the subcellular localization of AcERS1a, AcERS1b, AcETR2a, and AcETR2b *in vivo*, the ORFs without termination codons were fused in-frame with the GFP gene. By transient analysis using tobacco BY-2 protoplasts, the fluorescence of the AcERS1a, AcERS1b, AcETR2a, and AcETR2b proteins were localized in the cytoplasm (**Figure 8**), which is in agreement with the subcellular location of five *Arabidopsis* ethylene receptors in tobacco epidermal leaf cells (Grefen et al., 2008).

## Discussion

Unlike many other plants, pineapples can be forced to flower by the exogenous application of the gaseous hormone ethylene (or ethephon). This method has been widely used in pineapple production (Bartholomew et al., 2003), but the molecular mechanism of ethylene induction remains unclear.

Recent study of the evolution of ethylene receptors showed that in monocots, there are only two types of ethylene receptors: subfamily 1 receptors, which lack a receiver domain (S1-R or ERS1-like), and subfamily 2 receptors, which contain a receiver domain (S2+R or EIN4/ETR2-like), and ERS2 evolved specifically in the Brassicaceae (Gallie, 2015). In this study, we first designed five degenerate primers sets based on the alignments of the ethylene receptor sequences from other plants (**Supplementary Table S1**), and we attempted to cover all ethylene receptor genes in pineapple. Although five full-length cDNA clones were obtained by RT-PCR amplification and RACE extension, the sequence analysis and BLAST results showed that the cDNA clone from the AcETR1 primer set (**Supplementary Table S1**) belonged to *ERS1*, not *ETR1*. The cDNA clones from the AcERS2 primer set and the AcEIN4 primer set (**Supplementary Table S1**) were the same; both were typical undifferentiated ETR2/EIN4-type receptors found throughout monocots (**Figure 2**), (Gallie, 2015) and were more similar to ETR2 than EIN4. Therefore, these cDNA clones were designated *AcETR2b* (**Supplementary Figure S2**). We found there were only two ERS1s (Ac*ERS1a* and Ac*ERS1b*) and two ETR2s (*AcETR2a* and *AcETR2b*) but not other types of ethylene receptors in pineapple, which agreed with the results of Gallie (2015).

When the genomic DNAs of *AcERS1a* and *AcERS1b* were compared with their respective cDNAs, there were two introns located in the 5′ UTRs of the *AcERS1a* and *AcERS1b* genes. This result was similar to the result for the *AtERS1* gene, where a 236-bp long intron was located in the 5′ UTR, 29 bp from the start codon, and for the *CcETR1* gene, where a 978-bp long intron was located in the 5′ UTR (Bustamante-Porras et al., 2007).

The qRT-PCR results showed that the relative expression levels of *AcERS1b*, *AcETR2a*, and *AcETR2b* were significantly up-regulated (*p* < 0.05) after ethylene treatment (both at 200 and 1200 mg l^-1^) from 1–9 DAET and even at 14–21 DAET (**Figure 3**). From the histological results, BP growth developed at 9 DAET, and FP growth initiated at 21 DAET. By contrast, *AcERS1a*, *AcERS1b*, *AcETR2a*, and *AcETR2b* transcripts were initially detected mainly at the shoot apex in the early stages (0–9 DAET) and then in the BP and FP (14–28 DAET). When the flower formed at 37 DAF, these genes were mostly expressed in the flowers (**Figures 4–7**). The combined results of the qRT-PCRs and *in situ* hybridizations show that these four ethylene receptor genes play an important role during ethephon induction of pineapple flowering. However, to verify these genes’ function directly, transgenic pineapple or *Arabid*opsis plants and pineapple ethylene receptors knockout lines should be developed to study their flowering behavior, especially in response to ethylene, which should be addressed in a further study.

Ethylene receptors are negative regulators of the ethylene signal transduction pathway, that is, they inhibit the pathway when not bound to ethylene (Shakeel et al., 2013). Ethylene induces pineapple flowering, which means that the expression of ethylene receptors should be down-regulated in response to ethylene treatment. In our study, however, after treatment with ethephon, the relative expression of the pineapple ethylene receptors was not down-regulated but initially significantly up-regulated (**Figure 3**) and then decreases to the same level as the control. Ethylene treatment changed the expression of ethylene receptors differently for different plants. For example, the relative expressions of *OsERS2* (Yau et al., 2004), *LeETR4* and *LeETR6* (Kevany et al., 2007), *AdETR1* (Yin et al., 2008) and *DenERS1* (Thongkum et al., 2015) decreased after ethylene treatment, whereas the relative expressions of *AtERS1*, *AtERS2*, and *AtETR2* increased (Hua et al., 1998). The transcription of *AcETR1*, *AcEIN4* (Hua et al., 1998), *PhETR*1, and *PhETR2* (Dervinis et al., 2000) was not changed by ethylene treatment. RNA levels are not always positively correlated with protein levels. In tomato, for example, the expression of *NR*, *LeETR4* and *LeETR6* is low and constitutive throughout immature fruit development, and there is a significant increase in the transcription of *NR*, *LeETR4* and *LeETR6* at the onset of ripening. However, in contrast to mRNA expression, the receptor proteins levels were highest in immature fruits and significantly decreased to a lower level at the onset of ripening and thereafter because of protein degradation through the 26S proteasome-dependent pathway (Kevany et al., 2007). Therefore, whether the pineapple ethylene receptor protein is also degraded is the subject of the next study.

Pineapple natural flowering can occur under different environmental conditions, including low temperatures, short photoperiods, geotropic stimulation and other stress conditions (Bartholomew et al., 2003; Van de Poel et al., 2009). These environmental cues trigger ACC synthase gene expression, resulting in increased ACC production and ethylene biosynthesis (Botella et al., 2000; Trusov and Botella, 2006). In pineapple, ethylene acts as the signal for switching from the vegetative to the flowering stage (Burg and Burg, 1966; Min and Bartholomew, 1997); thus, these environmental cues induce pineapple flowering. Ethylene receptor genes can be induced by various abiotic stresses (Zhang et al., 1999; Zhang J. et al., 2001; Zhang J.S. et al., 2001; Xie et al., 2002; Tao et al., 2015). In this study, a higher concentration of ethephon might be perceived as one type of stress by pineapple seedlings, which resulted in the expression of ethylene receptors being sharply increased after ethephon treatment (**Figure 3**). On the other hand, our previous study showed that after ethephon application, the liberation of endogenous ethylene in both the shoot apex and D-leaf increased sharply until the 8th day when it reached the peak (Liu et al., 2011); thus, at a higher concentration of endogenous ethylene, more ethylene receptors were bound to ethylene, resulting in a gradual decrease of the transcription of the receptors (**Figure 3**). The reduction in ethylene receptor levels can increase the sensitization of plants to ethylene; subsequently, a lower concentration of ethylene can stimulate the ethylene response (Tieman et al., 2000; Cancel and Larsen, 2002; Wang et al., 2013a) and induce pineapple flowering. In this study, we found that the new BP emerged at 9 DAET (**Figures 4d**, **5c**, **6c**, and **7c**), immediately following (or at the same time as) the ethylene production peak.

*Cis*-elements play important roles in the regulation of gene expression by controlling the efficiency of promoters. In this study, a range of putative cis-regulatory elements that are responsive to ethylene, auxin, cytokinin, gibberellin, light, and abiotic stress was found (**Supplementary Table S4**). During the period of ethylene-induced pineapple flowering, ERELEE4, LECPLEACS2, and GCCCORE motifs were found in the promoters of these pineapple ethylene receptors (**Supplementary Table S4**), which suggests a potential regulation of these ethylene receptor genes by ethylene. In addition, among the *cis*-elements related to plant hormones, there were 10 different types of *cis*-elements involved in the auxin response (**Supplementary Table S4**). Because the shoot apex is one of the main sites of auxin synthesis (Normanly et al., 1993; Ljung et al., 2001), auxin may be involved in the expression of *AcERS1a*, *AcERS1b*, *AcETR2a*, and *AcETR2b.* On the other hand, there were multiple ARR1AT motifs in all four promoters (44, 36, 16, and 21 ARR1AT motifs in the promoters of AcERS1a, AcERS1b, *AcETR2a*, and *AcETR2b*, respectively; **Supplementary Table S4**). ARR1AT binds to *Arabidopsis* response regulator 1 (ARR1), which is involved in cytokinin signaling (Ross et al., 2004) and may be a link between cytokinin signaling and meristem regulation (Bao et al., 2009). By contrast, pineapple flowering originates from the shoot apex meristem (**Figures 4–7**), indicating that cytokinin may be involved in the regulation of the four ethylene receptors’ expression and further flower formation in pineapple.

In *Arabidopsis*, the five ethylene receptors (Grefen et al., 2008) and REVERSION-TO-ETHYLENE SENSITIVITY 1 (RTE1) are localized at the ER and the Golgi (Dong et al., 2008); our result of subcellular localization showed that AcERS1a, AcERS1b, AcETR2a, and AcETR2b were mainly localized to the cytoplasm (**Figure 8**). In addition, other important components of the ethylene signaling pathway, such as EIN2 (ETHYLENE INSENSITIVE 2) and CTR1 (constitutive triple response 1), are localized to the endoplasmic reticulum (ER; Gao et al., 2003; Bisson et al., 2009). At the membrane, CTR1, EIN2, and RTE1 regulate the ethylene receptors via physical interactions (Ma et al., 2014). Therefore, further studies should focus on these genes’ interactions with the ethylene receptors in pineapple.

## Conclusion

Our results showed that ethylene receptor genes (i.e., *AcERS1a*, *AcERS1b*, *AcETR2a*, and *AcETR2b*) may play an important role during inflorescence development in pineapple because the relative expression of these genes was significantly increased by ethephon treatment (**Figure 3**), and the expression was mainly focused in the newly formed BP, FP, and flower structures (**Figures 4–7**).

## Author Contributions

Y-HL and G-MS conceived and designed the experiments. Y-HL conducted experiments and performed the RT-PCR, quantitative real-time PCR, subcellular localization and *in situ* hybridization. Q-SW, S-HL, and H-NZ participated in the preparation of the plant material and the field experiments. XH participated in writing the manuscript and subcellular localization. ZZ performed part of the data analysis. All of authors in this study read and approved the manuscript.

## Conflict of Interest Statement

The authors declare that the research was conducted in the absence of any commercial or financial relationships that could be construed as a potential conflict of interest.

## Footnotes

1 http://www.ncbi.nlm.nih.gov

2 http://www.arabidopsis.org/

3 http://www.ncbi.nlm.nih.gov/Structure/cdd/cdd.shtml

4 http://www.expasy.org/tools/protparam.html

5 http://www.ebi.ac.uk/clustalw/

6 http://www.ch.embnet.org/software/BOX_form.html

7 http://www.dna.affrc.go.jp/PLACE/signalscan.html

## Supplementary material

FIGURE S1 **Ethylene receptors of pineapple**.

FIGURE S2 **Sequences of pineapple promoters**.

TABLE S1 **Oligonucleotide primers used in the experiments for isolate DNA fragments, rapid amplification of cDNA ends, full-length cDNA cloning and promoters isolation**.

TABLE S2 **Primers for quantitative real-time PCR and *in situ* hybridization**.

TABLE S3 **Primers for subcellular localization**.

TABLE S4 **Putative cis elements identified in AcERS1a, AcERS1b, AcETR2a and AcETR2b by PLACE. Numbers 1, 2, 3… represents the repeats of cis-elements while 0 indicate the absence of that particular cis-element**.

## Abbreviations

BP: bract primordial
DAET: days after ethylene treatment
EIN4: ETHYLENE INSENSITIVE 4
ERS1: ETHYLENE RESPONSE SENSOR 1
ERS2: ETHYLENE RESPONSE SENSOR 1
ETR1: ETHYLENE RESPONSE 1
ETR2: ETHYLENE RESPONSE 2
FP: flower primordia
GFP: green fluorescent protein
MW: molecular weight
pI: isoelectric point
PLACE: plant *cis*-acting regulatory DNA elements
qRT-PCR: quantitative RT-PCR
RACE: rapid amplification of cDNA ends
TAIR: The Arabidopsis information resource; UTRs, untranslated regions
