# Supplementary figures and images for: Molecular Cloning and Characterization of Four Genes Encoding Ethylene Receptors Associated with Pineapple (Ananas comosus L.) Flowering
Source: Front Plant Sci. 2016 May 24;7:710. doi: 10.3389/fpls.2016.00710 (PMC4878293; doi:10.3389/fpls.2016.00710)

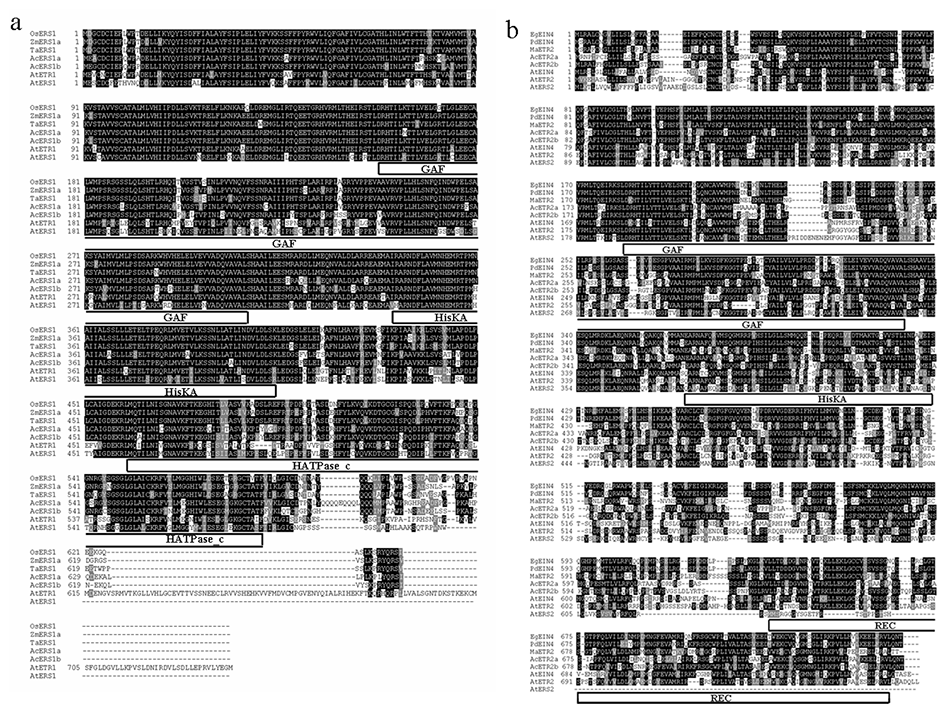

Supplement: FIGURE S1 — Ethylene receptors of pineapple. [file Image_1.TIF]
