# Supplementary material for: Molecular Cloning and Characterization of Four Genes Encoding Ethylene Receptors Associated with Pineapple (Ananas comosus L.) Flowering
Source: Front Plant Sci. 2016 May 24;7:710. doi: 10.3389/fpls.2016.00710 (PMC4878293; doi:10.3389/fpls.2016.00710)
Supplement: FIGURE S2 — Sequences of pineapple promoters. [file Image_2.PDF]

The *cis*-elements involved in the response to ethylene predicted by PLACE software are indicated in red letters. The sequences of the genes are shown in italic letters, putative start codon (ATG) is labeled with a rectangle.

>*AcERS1a* promoter

GCTTTCCTAAGCTCCAAGCGAACAACAAATTAAATGTGAAAAACGAAAAAAAAAAAACTACTGCTA
GAAGTAGAGCTATAAGGAATCGAACCTTCTATTCTATTGCGCTCTCAATTCAACAGGTGGAAGGTCA
ATAACCCTACAAAATTTTATTGAAGTACATGCATTCAATTGTAGTTTTATTGGGAGATGAGGAGGAAA
CGGTTTTGGAAGATGAGGGGTGTTGGAGGTTGGGAGATGAGAGAGAATAGTTTTTGTTCAATTATAT
ACATTCAATTGTGGTTTTATTGGGAGATGAGGAGGGAACGGTTTTAGAAGATGAGAGGTTTTGGAG
GTTGGAAGATAAAAGGGAGGAGTTTTGAAAGAAGGAGGTAATGCCATGTCAGCAAGATATTGGGGA
TTCATAGATTAGTATAAATTGGGCCGCTCTTGATGTGTGGATATACTAAGTTTTTAAAATTATTTAATTT
TAAATAGGCCAATTTGCATAAAAAAATTTATAAATTTTGAGCTTTTACAAGACCGGGCCGTCTTTTTTT
ATTTTACAGATTCGGTCCGAATTTTCAACAAATTGACAAAAATACCCTAATTACTTTCTTTCTCTCCTT
TTTCTCTCTCCGCTTATTTTCTCTCGTTCATTTTTTTTTTCTCGCCGAAGAAAGCAACAGAGGCGGAG
GTAGAGGCAGCGACGAAAACAACCCGCCTCGCCTCTCACCCTCATGCTCTCGCCCTCTCCCTTGCC
CTCGTCCGCGTACCCCCCACCTTCCTCGCCTTCGACCCCGCCGAGGCCGCACCGCGACTCTTGTTTT
TTTTTTTTTTTCCTCTCCGACTCTTCTCCACCGTTTTCGACGACGACGCATCTTCGCTGACGCCGACG
CCGATACCGTGAAGGTCGCTGTAGCGCTGCAACCTCGGAGCGGACGGTTCTTAGCGGCGTCGTCGC
AGGCGTCGTGATCGTTGGCAGAGAGGGGTGACAAAAAAAATTATAGAAGAAGTAAGAAAAAAAAA
TAAAGATAAAAAATTATAGGAAGAAAAAAGATGAAGTTGAAATTGTACCGTTAAAAAAATGTTGCA
CTATTATGGATATAAGTTGCACTACTTAAGATATAAACTACAGCTTTAAGATAAAAATTACACTCTTTG
AAAGATATTTACACTGTTTTTCTTCTTATTGTACTCTTTCAGATATAAACTGTCTCTATTAAGAAAAAA
GTTAAACTCTTTAAACGAAAATTATACTTTTTGAGAGATATGTATACTGTTTTTTTTTCTTGCACTCTT
TGAGATGTAAAATACATTCCTTTAAGAGATATTTATACTGTTTCTTCTCTTGTTATATTATTTTTTCTCTT
GTTGAACTATTTCTTCTCTTATTACATTGTTTCTTCTTTTAAATGGTGCAGTTTAAGATAAGAAACAGT
ATAACAAGATGAGAAATAGTACAATAAGAGGAGAAACAATACAACAAGAAGAGAAACAGTATAAAT
ATCTCTTAAAGGGCTGCAATTTACATATTAAAGAGTGCAACAAGACGAGAAATAGTGTAAATATCTC

TCAAAGAGTGTAACTTTCGTTTAGGGTATGTTTAGTTGTCCGTAAGTGATCGAAGTGAAAAAAGTCA

CTATTCAAAAAGTGACTTTTATAAAAGTCACTTTTGAGTTTAAGTGTTTGGCTTCAAAAAAGTAAAA

GAAAAAAAAAATATGGCTTTTATGATTTCTATTGTTTGGTTCTATATAAGAAAATGAAAAAAAAAAA

ATCTTAGATGTGTAGTCTTTTTCTTTTCTTTTTTTTTTCTTTTTTTTTTTTTTTTGTAGATGGTATGGAAT

AAGGTACGTCTCTTTCTTTGTTTTTTTTTTTTTTTTTTTAAGAGGTGTTGGTTTTTTTGTTAAAATATG

LECPLEACS2

GTGATTTTTTTTAAAAAATTTTTGTAGATGGTATGGAAGAAGATTTTTGGATACGGTTCCTAAGTTGT

GTGGAGTAGGCGGCCCACTTTCGACGACTTCGGAATTTTTTTTTTCGAAGTCAAAAAAGTGGATTTT

GCCCORE

AGTTATAATCCACTTTATCGGTTCACTTTTCAAAAGTCACTTACATATTTACTTTTATAATCCAAACAA

TCGAAATAATCCTACTTTTTCAAAATACCACTTTTATTGTTTCACTTACAGGCAACCAAACACGCCCT

TAAGGAGTGTAACTTTCATCTTAATGGATACTCTTTACATCTGAAAGAGTGCAATAAGAGAAAAAAA

ATGTAAATATCTTCTAAAGAGTGTAATTTTTATTTAAAGAGTGTAATTTTTATCTTAAAGCTGCAGTTT

ATATTTTAAGTAGTGCAACGTATATCCATAATAGTGCAACGTTCTTTTAAAATAGTGCAATTTTATTTT

LECPLEACS2

AAAGGTGCAATTTCATCTGTATCTTTATTTTTTAATAATTTTTCATCTTTATTTTTTTTCTTGTTTCTTCT

ATAATTTTTTTTTTTGTCACCCGTCTCTGCCAACGGCCACGGCGCCGGCGACGACGCCGCTAAGGAC

CCCACGCTTCGAGACTGCAGCGCCGCAGCGACCTCCACGGTATCAGCGTCGGCGAAGATACGTCGT

CGTCGGATGCGCCAGAGGAGGAGGGAGAGGTAGAGGACGAGAAGGGCAGGGAAGGGCGAGAGA

GGCGTCGTCGTCGGAGACGGTAGACTAGAGTCGGAGAGGAAACAAAAAAGAGTCGCGGCGCGGC

CTTGGCGGGGTGGGAGGCTAGGAAGGTGGAGGGTGCGAGGGCGAGGGCATGAGAGTGAGAGGCT

AGACGGGCCGTCTCCAGTGGAGGCAGAGGAAAAAAATAATGAACTTGAGAAAAAAAAAAGAGAA

AGAGGAGAGAGAAAAAATAATAAAGGTATTTTAGTTAATTCGCTGAAAATTCAGTTTGAATCTTATC

AAATTGAAAAAAGTAGCCCACTTTTATAAAAGCGCAAAACTAGTGGATTTTTTATGCAAATTAGCCT

TTTAAATACGTAACTATTTTAGAAAACCGAATTTTGTGCCGAGCGAGAACAAATTATGCGGTTCGGT

TCATGGATGAACGCATGGATCAGGTCCGCGCAACAATTTCCCCACTTTTTTTTTTGTTTTTGGTAATA

CGCCACGATAAACTCGCCAAAAAGCTTCCCCAGTGCTGTAGCTGCAAAGCGCTCTCTCTCTCTCTCT

CTCTCTCTCTCTCTACTCTTTCCCTTTCCAACTCTCTCTCTCTCTAAGTGGACTCCGTGGAGATCGAG

CCGACGAAAACCATGACGCCATGGGCGAGCTCCTCCTCTGCAGCGATCTCCGATCCCCAAGTAGCT

TCTCTCTTTAGAGTGAGAATCTCTCACTTCGATCCTCTAATTGCTTCTTTATCGGTCGATTCTCTTGTT

TAATCGCTCGTAGTTTTTTATTTTAGTGTATTTAGCTCGGTGGAGATTGCTTATGAGCTTAGATCTGAT

CTTCTGTGGTTTTTTTTTTTTTGTGGATTTTTTGGTGATTATTTAGTGGATTTAGGGCTTTTTCTTCTGGT

GTAAGAATCGGAGGGGACGATTCTTCTGGTGTAAGAATCGGAGGGGACGATTTCGGTGGAGTAGAG

ACGATTCGATGTTTGGGATGTTCACTGATCGGTTCTAGAGTTTCGGGCATTTAATTCGAGCTTAGTTT

GTCGATTGGGTTCTTTGAAATTGGAAAAAATTGGGTTAACCAATGCGTGTTTGTAGTTAATTTGCTA

ERELEE4

AAAGGGTACCTATTTTTTTTTATGGTATACTTGTGATCTTGCGAAATAGGATAATCTAATCTTAATAAG

CTCAAACTGGGAAAAGGGTTAGGTGGAGGATGGGGTGCCCAGAAGCAGAAGCGCTGCTGAAGCTT

TTGACCTGTGCTTTGGAAGCCAAAATGAGCTTTGTGATTTGTAGGAAAAAGCTGTTAGCCTTTAGCT

GTAAATTGCGACAAGTTTACCAAATTGAGTAACATTGTTCAAGTTCACTTAGGAGCTAATGATGGAG

GAAGGGATTATGAAATGGAGAAATAAGATGCAAAAGTGCTCGGATGGGAGTTGGATGGCCTGATGT

AGGAATTAACAAGAGATTTTGATCGAAAAAACCCATAATCTCATTGGCCTCCAAATAGATATCGTAC

ATTTTTGTCGTCGGTTACTCTTTTTTATTTCTTTCAATTTTATTTTTTCTCCGGAATAGGTGGTGTTTCC

TTTGAGCTTGACTTGTCCCAAGGCCGTCTTCTGGTTTGATAATAGTACTGAGGGCTGGAAAAGAAAT

TATGTCAGGAATTACGGTTTCTTGAATTGGCATGAATGCTGAGATGAGGACCATTCCAATGATTTGCC

TGATAGATGCTTCAAGAAACTATGTACCAAGGCGCTACCATTAAAAATCCGAAACCTGTTGCAGTTT

ATATGTATGATTAAGAATATGGAAGGCATAGCTTCTTCTTGAGTTGTTTGGTCAGTATTTAGGGACTC

ACTGGAAGAGTAGATTTGAGATCTTGTTATTGGTATTTTATGAACTCCTAATCATTTTTATGTTTAGGG

TCTAACAATTACTAAAAGTTTTATCTTTGAGTACTAGGAAATTGATTAGCTTTATTGCATTCCATCAAA

TTGCAAATCTTTTGTTTTTCTACATTATAAGATATTAGGGTTTGTCTTTCTCCCACTGCCCATGCCCTAT

ACTTTATATTATTTTTGTGATCCTCTATGCCTTAAATGTTTGAGTGACCTACCCCTTTCCCTCGCAAGT

TCTAAGCCGGTAGGACGCCTTAAGGAGTAAGTACTAACACATCTATTCGGAAAAAGGATAAACACA

ATGTAGTTGTGATTGCTCAGTTTGTGATTGGAAGAAGTCATAATGAAGAAACAATTTTGTTTTTGGA

CGGCAATATGAGCATGGTATTTGTCTGGAGATCTTTGGGAAATTATTTCTTCTTGCTAATTTTTCTTCA

CACTGTCTTTCAACTACAACTGTATTTTCCTTGCTTTGGTCTCTTCTTTATAATCTCATGAAAGTAGTT

CAGCTAAATAAAGTTCATTTATTTGCTTGGTGCCGACTCTTCTTTGTGTGTATGTGTGTGTGTGTGTG

CGCGCGCAGGAATGTAGAAATAAGTTTTTTCCTCTGTAATTACTGCTAA*ATGGAGGCTTGTGACTGCA*

T

> *AcERS1b* promoter

CAAAATCTCGATCGCAAGAACAACCGATGTACCTCCTCTTTCCTGATCTCAAACACGGTAACAATAA

GAAACTCCCCTTCGCAGGGCTCCACGCTCAAGCTCGACACTTCCTGAAACCTCTCCCAATTCCCCG

ATCAGATCCAAACAAGATAAACCCTAACCCAAAAGCTCCCAAATCGGAATCGAATAGTTCCAAATC

TCACCCCGGTTTCCTCATTGGCGATCCTGCGCTCGAAGAAGATCGGGGCCACGTGGGCGAAGACGC

GGCGGAACCCGCGGAGCACTCCGATCCTGAAGTTCTTGAGATCGGGGAACGTGCTCCTCGCGCTCC

TCTCTAGCAGTAGGAGAGAGGCGTTAATGGAGATCGGAGAGCTAGGGTTTCGTGCGAGAGAGAGA

GGAGGAGAGAGAGAAGAGAGAAGAAGAGGGGGCGGTGAGGTTACCGGAGAGGAGGGAGCCGAA

GCCGCATATGGATATATACCCTTCGGGAGTGACGAGCGAGTCGAAATCGGATGCGGTGTTGAGCTCG

GTGAGGTTACCGCTCGGTGCGCCGGAGTGTTCCGTAGCCATGCGGCGGAGCAGCGGAGGCGGTGA

GGTTACCGGCGGTGACCGGATCCCGTAGGAGTGAGCGATGCTGGAGCGGAGTGGAAGGAAGAAGA

CACAGGGGAGGGGTCGATGGGCGTCGGATCTCGATCGGACGGTCGGGGATGACCCGGCGTAGGAT

GCGAAGAGGTTTATAGCGGCGTGCATGGTTTGCATAAATAAATGATAACGGAGAGCCGGGCTTTTCT

ACGACCCACTTTGTTTTGTTAAAAAAGCAAGTGTTAAAAATTACACGTGGCAACTTCTCGTTGGCTG

TGAAATTTACGCTACGTCACTTTTGATATTTTTGTTCCGAGAATTAAATCAAAAAAGTTATATTAAAA

AGCTCATCGCAAGCATTTAAAAAAAAAAAAAAAAAATGATAGTCCCAATGTGTACATTGTAGTTTATC

TATTGTAACCATCTAAAAATATTGTTTTATAAGATAAAATGATTTGCTTATTTATATAATTATATTACTGG

GATAAAATTAAGAGATCATTTCATAAAAATTATTTAAACATTAATTTTTTCTTCATTAGTTAGGAACTA

GAGTATTATCAATATATCTGTATTATTTGTAGAGTAACTATTTTTATTGCGTCGAGAAAATTCTATTCTT

TTCCGATAACAGACGCAAAATAATAAAAGTATCATATCCGATATTCGGAATTGATTGATGTACACAAT

TTTGTTTTCAATAGATGATAGATGATGAAGTTGTATGCTTTTTTTTATAAAACTCTTAGCATCAATGAT

TGCTATAGTTTTTTTGTTTAAGTAATATTTAAAGTTACCGCGAGGCGTGGATCATGGATGAGTTTATAA

TAGGATTCAAATAAATTATTTGTATAGGTATCCTAATAAAATTTGGGAGAAAAGGAACAATCCTTTCC

CCCACAATTTATATGGAAAACGCAGATTTTATACTACTGTAGTAGAATCTTAGAGTAAGATTACATAA

ATACAGTACAAACAAGGACAATTATTATTATATAAATAAAAATAATAATACACATTATAGATTTGCATG

CCATATATAATACGTAAATAATTGAGGACTTAGGTGCAAATATTCCATTAAGTACCTGAATATTTGGGC

CATTAATGAGTTAACAGCATTTTTTTTTCTTTTTTTGGGGTTTAAATCATTATTTTTTCTTAATATTCTA

GTAGTTAAAATTGTAAAATATAACAAGTAATATATTATTATATAATGACCTGTTTACTAATTGGCCAAG

LECPLEACS2

TAATTAATCCTTCAATTTATTACATAAATGAATATTTAATTAAATATTTAAGCAATTAATTTTTTATTTAT

AATTAACTAGCTAACTAAATAATTAAATGTGTTACGTGCTCATTCATTAAAAAGTTTCCATATTAATTA

AATAATAACTAATATTTTGACTAATATAATTTAATACTTGAGCTAAGCGTAGCCAATTAACTAATTAAT

GTGCTGCTATATTATCCTGTACGATATTCATAATTAGTTAATTAATTAAGATTTTAAATTATTATTTTTTA

AATTTATTTAAAATTTCTAATTACTTCTATTCCCACAAAGCTAGTGTAGCCATTCAAAGGCTATTTGAT

TTTTTTTTATTACTCAGAAATTTTAATTCTGGCCCAAAAATAAAATTAATCTAAATTTTTTTATTTAAAAT

LECPLEACS2

ATTTTGGATGTATTAAAAAATTAAGAATATTTTTTTTCTGAATCAAATAATATGGAAGGAGTTTAGGGA

TCAAAGTGAAAAATTTAATCCTTTTGACGACACTAACACATGCTTTATCTCTGATATGCTTTAAACTC

GGAGCGTGGCGAAGCTTCTCGTCTCCGCGGCGGCGAATCGAAAGACTCCAAAAAAGATTTTAATTT

GCCCORE

TATTTTCTCGTCTCTTTTTCGCTCTCCTCCGACTCTCTCTCTCTCTCTCTCTCTCTCTCTCTAACCACG

TTACTACTCCGGAGGAGTCGGATTCGGCCTCGTTCGCCTGCGAATCGCTTGATCACACCTCCATCGA

AGAGTACATCTTTCGCTCTCTCGATCTTTCCACTGCTTAATTTTGCTGTTTGATGCTCCTGATTCTTTT

TTTCTGTTTTTGAATGAGCTTAGATCTGGTGAATGAAGGGAATATTGTGAAATTTGGGGTTTTGTGG

ACTCTGGTGTGTTCTAGGGTTCTGGTGTAGTTGATTTGGAGCCCCATTTCTCGACTTCGTTATGTGGA

TGGGGGGAAGTCGAGAGATTGGAGGTGTTTTTGAGCTAAAAATTAGAGTTTTTTTTTCTTCTTCTTT

CTGTGTTTTTGAAGTGATCAGGTTCAATTGGGAAAAGTATTTTGTGCAGGATTATGTTGTTTGAGATG

ATTGCTAAGCTTTGGATAGTGAATTGTTGAGATTTGGCTAGATGCATCGCTTGATTATTGGTTGTGTG

AAATGTAGATCACATACTTTGTATTATTTGGGGAGAACTAGATAGAAGAAATGCGAAATAGTTGATGT

TATTGAGGATCCCTTGTGATGGTGTGCTTTTTTTCCCCCTATTTATATTTACATTTTTACTCTTGTTTTATT

ATGTAGAATGTTTTCCTCTGATAAATCTTGCTGAAAATGCTGCAGTAATTCGTTATATGTGCTTCCTTA

TGACGAAACTAATTGGATTTCTACCTGGTTATTTTCTTTCTCACTGTCACTCTTAACTATCAGTTCAA

CTTATCTATGAGGCTTTTAGTAGTTTGTGACTTCCATTTGAAGAATAGTGCATCCCTTTATCGTGCTTA

GCATACTATTCTTACAAGCATCCTATTAAATTGCTTGTAGCGCTATGCCCTCTTTGTATCGAAATTAGA

AGCGAGAGGAGTAACCAAACTTCATGTAGGTTGGTATTGACATTAAGTGTTTCTTCTTGCCAGGAAT

GTAGAGAAAAATTATTGTTGCTGACAATCACAAGATG*ATGGAGGGCTGTGATTGTAT*

>*AcETR2a* promoter

ATTGAAGAGCAGGGAGAGGAGGATGAGGAAGAAACCCTAGGAGCGGGGGAGAGGATAAGGAGGA

GGAGGAGAGTGGAGTGGGACGATGGAGAGCGAGAAGCGGAGACATGGGGTGGGGCGAGGGAGA

GGGGAAATAGAAGAAGAAGAAGAAGAAGAAGAGGCGTTGTCGTCGGAGATGGTGGAGAAGA

GTCGGAGAAGAAAAGAAAAGAAAAAAGCAAAAAAAAAAGGTCGCGGCGCCGCCTTAACGGGGTC

GCCCORE

GCAGGCGAGGAAGGTGGGGGGTGCATGGACGAGGGCAAGGGCGAGGGCGAGATCATGACGGCGA

GAGGCGAGACGGACCGTTTCTGCCTCCGCTCCGCCTCCGCTCTACCGGCGAGAAGAAAAAAAAAG

AACGAGAGAAACGAAAAAGAAAGAGAAAGAGGAAAGAGAAGAAGTAATAAGGGTATTTTGGTCA

GTTTGCTGAAAAAGCGGACCGAATCTTCAAAAATGAAAAAGGTGGCCCGATTTTGCAAAAGCCCA

AAATAAGTAGATTTTTTATGCAAATTCGCCAAAAAAAAAAGTGTTGGATAGTCTTTATAATTAAGCATA

AAAATATAAATGTTAAAGGGTGATTTTCGAAACGTTCTACAGTTGAGGGGGTTTCCATAAGAGGTTT

AAAAAAGGGACCTCCTCCTCTTGCAATGCAAAGCAACTAAGTAATAATGCTAAGCGAAGCCTCTCT

CTCTCTCTCTCGCTCTCTCCTCTTGTTTTTGATTTGTTCAATGTTTAATTTCTTGGTATTTTTAATGTTT

GAGGGATTTATGTGCAAGGAGTGGGCAAAGGAGTGAACTTTTTTTTTGAGAAAGAGTGGATTTTAA

ATTTATTTTGTCTTGTTTTGTTGTTAATTTTTGAGTTTTCTGAGTCTTAAAATGGCTAAAAAGGTGAG

CTTTTTGGACCAAAAAAAGAATGTTTTAGGGAGTTGTTGAAGTTATTTTGATTTGTTTTATTGTTAAT

TATTGAGGTTTAAGTCCAAAGAGGGTCAAAAAAAGGGAAAAAAGTGAGCTTTTTTGAGGTTATTTT

GATTTTTGAGGATCCAATTAAGTAGAAAGAGATAGAAAAGGATTGAGCTTCTTTTTTTTTTTAGCAG

GGAATTAATGGTTTTTTCTTGGGTTTTTAAGTTGGAAGAGGTAAAAAAGAAGTGAGCTTTTTTAACA

ATGATTTTCCTTTTTCTTTTGTTGTTAATCCTCGAATACTCATTGCTATTTAGAAAGAGAATTTGAAAA

GCAAGGAGTGGATTCTGAGTTTCTTGTGTTGAGAGATGTGATCTTTTCCTCTATATTTGGGTAATATT

TTTTTAAAAAAAACAAAAATCCTTCCCTCAAAATACCAACGAAGGAGATCAAAATGCCCCTCTTTTC

ATTTCTTTTTTTAAAAAAAATCATAGTCATGCACTAGAACTCCTCTTCAAGCAGCTGTTAGAAGTGTT

CTTCTTTCAATGATCCTTCTTACTGTTCCTTTTCTCATTTGATTCTTTTATATATATATATGTAGTTAGAT

GTTACAAGTAATGATAATGATGATGATGATGATGATGATGATGATGATGGCTCTTTGATCCAA*ATGCG*

AAATCCAATCCCCTG

> *AcETR2b* promoter

GTCCTCAAGCGGCCGCTTCTTAGCGCGGAGGTGAAAAGGAGAGAGGCCCAGTTCCCCCTTTTGGG

CTCCCCTGATTCAAATCAGGTGGATCTCATGGTTGCTTTGGGAGTTTTTGGTGTGAAAAACGTGGAT

TAGAGATGGTGGTTTTGTAGGTAGTGGTTTTTTTTCCGAATCCCCCCTTGGTGTTGCTGTTTTTGTTG

GTGGTTGGTGCTCCTGTGCCTCTCCTGTTGATTATTATTATCATTTTTTTTTGCGCTTAAAATGGTTGG

GTTTTGTGGCTATTTGGGTGTATAAAAGGCTGGGGAAGGATTAAGTAAAGGTGAGATTTTTATGGTG

TTGGGATCTTATTTACAGGTTTGGTGTTAAAGGGCATGTTGGATTTGATAACTCTGTGGCACGATTTG

TTGTGTTTGATGCTACTAAAACATGTTTAATACATGATCCTGAAGAAATTATGGCTTTTTTTGGTGATG

TTTTGGTCCATTAGAAGAGGAAAGAAGTGATTTTGGGCTTTAGGGGTTTAGTTTTATTTATTTACTGT

TTGTGTGAAACTTAGTTCTCACATTTAATGCTCAGAAATTTTCTCATGCTGTTTTATCCTTCTTTTGCA

TGCTGATTAGGGCTAGTATGTTCCTATCTTGCTGGAATCTCTAGAGGATCCCCGGGTACCGAGCTCGA

ATTCGTAATCATGGTCATAGCTGTTTCCTGTGTGAAATTGTTATCCGCTCACAATTCCACACAACATA

CGAGCCGGAAGCATAAAGTGTAAAGCCTGGGGTGCCTAATGAGTGAGCTAACTCACATTAATTGCG

TTGCGCTCACTGCCCGCTTTCCAGTCGGGAAACCTGTCGTGCCAGCTGCATTAATGAATCGGCTTAG

ATCTTAGATAATTATATAGATTTGTAAGTTGGTTTTATTTTTAGATCTTGTAAATTGCATCTGAATGGAT

TATCATGCCTGGTATATTTAAGCAGATGATTCTATTCATGTTGTTAAAATATGTTCTCTTTTGCTCTTAA

AGTTTCTTTCTTGTAATCTTAGTTGTGGCGATCCATCTGTAATCTTGTTCTAGGAAGATAAAGAGTTT

TGATTTCTGCGATGGCGCATACACGAATGGATTGAATGGATGAAGCTTTGTAAGCCCCCCAA*ATGTTA*

AGAGCACTGTTCCA
