# Supplementary material for: Molecular Cloning and Characterization of Four Genes Encoding Ethylene Receptors Associated with Pineapple (Ananas comosus L.) Flowering
Source: Front Plant Sci. 2016 May 24;7:710. doi: 10.3389/fpls.2016.00710 (PMC4878293; doi:10.3389/fpls.2016.00710)
Supplement: TABLE S1 — Oligonucleotide primers used in the experiments for isolate DNA fragments, rapid amplification of cDNA ends, full-length cDNA cloning and promoters isolation. [file Table_1.DOC]

**Supplementary table** 1 Oligonucleotide primers used in the experiments for isolate DNA fragments, rapid amplification of cDNA ends, full-length cDNA cloning and promoters isolation

| Primer name | Nucleotide Sequence (5’- 3’) |
| --- | --- |
| AcETR1F | CACCCACCTGATCAACytntggacntt |
| AcETR1R | GGGTCCGCATCTCGTGRTTCATNAC |
| AcERS1F | GCCCTGATGCTGGTGcayathathcc |
| AcERS1R | GGTTCATCACGGCCARRAARTCRTT |
| AcETR2F | ATCCGGAAGTCCCTGgaymgncayac |
| AcETR2R | GGTCACGCTGCAGCCNARYTTYTC |
| AcEIN4F | AGTTCATCGCCTTCATCgtnytntgygg |
| AcEIN4R | GATCCAGATGTTGCCCTBCATCATYTG |
| AcERS2F | TGCTGTTCCAGTTCATCgcnttyathgt |
| AcERS2R | CCTCCGGGGTGCCNCCYTTRAART |
| AcETR1SP1 | AGCTGTTGCACAAGACACGACAGCA |
| AcETR1SP2 | GCCAGGGTTTTCGAGTGCATGTT |
| AcERS1SP1 | TCCGGTGGAACATACCTCCCCACT |
| AcERS1SP2 | CAAGACCCAAAGTTCTCCCCAGTTC |
| AcETR2SP1 | CGGCTTCTTTGACCAGCGATTTGAG |
| AcETR2SP2 | CGTAGCACGCCTCGATCACC |
| AcEIN4SP1 | GCTTGCCTCCTCCTGCCTCTTCATC |
| AcEIN4SP2 | AGGGTTATCGCAGTGGCAAAGG |
| AcERS2SP1 | ATGGTGTGGCGGTCGAGGGACTT |
| AcERS2SP2 | GCAGAGGACGATGAAGGAGGTGAA |
| AcETR1SP3 | GCAGATATGTCCCGCCCGAGGTAG |
| AcETR1SP4 | CAGCTCGAAGGGAAGCCGAAA |
| AcERS1SP3 | CCCGTCGGATAGTGCGAGGAAATG |
| AcERS1SP4 | GCTCGGCAGGAGGCAGAAATG |
| AcETR2SP3 | CCGAACAGCGAGGGGTTTGATATGG |
| AcETR2SP4 | CGAAAGCTCCTCGGGAAACTGG |
| AcEIN4SP3 | GCGAGGCGAGGCAAGATCAAGAATG |
| AcEIN4SP4 | GGAGATGGGCCTTAGCTTCAGCA |
| AcERS2SP3 | CGCCAAGGTGCTCAATTCCAAGTCC |
| AcERS2SP4 | CGAATGCCGATGCTCAAAGTCTC |
| AcERS1aSP5 | GTGGAGATCGAGCCGACGAAAAC |
| AcERS1aSP6 | CCACAATGCCGATAACTCGTCCA |
| AcERS1bSP5 | CCTGCGAATCTCTCGATCACACCT |
| AcERS1bSP6 | GGCCTGCGCAACTTCATTTACAAC |
| AcETR2aSP5 | GGGGATGATGATGATGATGATGGTT |
| AcETR2aSP6 | GGAACCAAAACTGCTACGCAAAAGA |
| AcETR2bSP5 | GGTGTTGCTGTTTTTGTTGGTGGA |
| AcETR2bSP6 | CCGGTAAAAAGCCCCATAGAGCAG |
| AcERS1a-GSP1 | TCAGTGAACATCCCAAACATCGAATCGTC |
| AcERS1a-GSP2 | AATCGTCCCCTCCGATTCTTACACCAGAAG |
| AcERS1a-GSP3 | TCTAAAGAGAGAAGCTACNNNNNNNNCGGAGA |
| AcERS1b-GSP1 | AGAACACACCAGAGTCCACAAAACCCCAAA |
| AcERS1b-GSP2 | AGCAGTGGAAAGATCGAGAGAGCGAAAGATG |
| AcETR2a-GSP1 | GAGACCAACGCGGTGAAGAACTTGGAGA |
| AcETR2a-GSP2 | AAGTAGAGCAGTTCGAGCGGGATGGAGAAG |
| AcETR2b-GSP1 | AGTCGCTCACTTTTTGGCACTGGAGGATG |
| AcETR2b-GSP2 | GCGGAGGTGGAGAAGAGGAGGGAAAGGA |
| SFP1 | CACGACACGCTACTCAACAC |
| SFP2 | ACTCAACACACCACCTCGCACAGC |
| SiteFinder1 | CACGACACGCTACTCAACACACCACCTCGCACAGCGTCCT  CAAGCGGCCGCNNNNNNGCCT |
| SiteFinder2 | CACGACACGCTACTCAACACACCACCTCGCACAGCGTCCT  CAAGCGGCCGCNNNNNNGCGC |
