# Supplementary material for: Molecular Cloning and Characterization of Four Genes Encoding Ethylene Receptors Associated with Pineapple (Ananas comosus L.) Flowering
Source: Front Plant Sci. 2016 May 24;7:710. doi: 10.3389/fpls.2016.00710 (PMC4878293; doi:10.3389/fpls.2016.00710)
Supplement: TABLE S2 — Primers for quantitative real-time PCR and in situ hybridization. [file Table_2.DOC]

**Supplementary table 2 Primers for quantitative real-time PCR and *in situ*** hybridization.

| gene | Forward primers (5′–3′) | Reserve primers (5′–3′) |
| --- | --- | --- |
| **for quantitative real-time PCR** | | |
| *AcERS1a* | TGGAGGCCGTATTGAAGAG | GCACAATGGCAAATCTGGT |
| *AcERS1b* | GCTGCACTCATCAACGATGT | CACAGTGGCAAGTCTGGTGT |
| *AcETR2a* | GAAATTCGAGATCGGGGTTAG | GAAGCGAAGGACGAGAGTCAT |
| *AcETR2b* | TTCTCGGAAACCCTCAGAAA | CCCTTGAAATTAGGGGAGGA |
| *Actin* | CTGGCCTACGTGGCACTTGACTT | CACTTCTGGGCAGCGGAACCTTT |
| **for *in situ* hybridization** | | |
| *AcERS1a* | GCCGTATTGAAGAGCAGCA | GTCCGGCTTCACAACAGAA |
| *AcERS1b* | CAAAGGAAGGCCACATTTCA | CTCTTCGTCGGCACTATTCG |
| *AcETR2a* | GAGAAGCCGAAGAATTGCAG | TTGACGAGAGAGGAGGAGGA |
| *AcETR2b* | CGAGTTGTTGGTGACGAGAA | GTGGCCTTTGGAGTTTGAAG |
