# Supplementary material for: Molecular Cloning and Characterization of Four Genes Encoding Ethylene Receptors Associated with Pineapple (Ananas comosus L.) Flowering
Source: Front Plant Sci. 2016 May 24;7:710. doi: 10.3389/fpls.2016.00710 (PMC4878293; doi:10.3389/fpls.2016.00710)
Supplement: TABLE S3 — Primers for subcellular localization. [file Table_3.DOC]

Supplementary table 3 Primers for subcellular localization

| gene | Forward primers (5′–3′) | Reserve primers (5′–3′) |
| --- | --- | --- |
| *AcERS1a* | TCTAGAGGATCTCGAGATGGAGGCTTGTGACTGCATAG | TCTCCTTTACCCATGGTACC TATGCTCCTTTGATACAGGGGTT |
| *AcERS1b* | TCTAGAGGATCTCGAGATGATGGAGGGCTGTGATTGT | TCTCCTTTACCCATGGTACCTATACTCCTCTGATACCGGGGC |
| *AcETR2a* | TCTAGAGGATCTCGAGATGCGAAATCCAATCCCCTGT | TCTCCTTTACCCATGGTACCTGTGTTTTGAAGAACCCTGATGA |
| *AcETR2b* | TCTAGAGGATCTCGAGATGTTAAGAGCACTGTTCCATGGGC | TCTCCTTTACCCATGGTACC CGTGTTCTGGAGCACTCTGT |
